# From histological recovery to residual gastric cancer risk after *Helicobacter pylori* eradication: a multi-database bibliometric and clinical decision-domain analysis

**DOI:** 10.3389/fmed.2026.1962890

**Published:** 2026-09-23

**Authors:** Houshu Tu, Menglin Chen, Panpan Zhu, Jing Hong, Ling He

**Affiliations:** 1 Jiangxi University of Chinese Medicine, Nanchang, China; 2 Affiliated Hospital of Jiangxi University of Chinese Medicine, Nanchang, China

**Keywords:** bibliometrics, chronic atrophic gastritis, endoscopic surveillance, gastric cancer, *Helicobacter pylori*, residual risk, risk stratification

## Abstract

**Background:**

Successful *Helicobacter pylori* eradication reduces gastric cancer risk, but residual risk persists in patients with established atrophy or intestinal metaplasia. We integrated multi-database science mapping with independent article-level clinical decision-domain classification to quantify how the research agenda evolved from mucosal recovery to residual-risk management and to identify decision-critical evidence gaps.

**Methods:**

Web of Science, Scopus, and PubMed were searched through 27 July 2026. Records were deterministically deduplicated and independently screened. Bibliometric networks, thematic structure, temporal bursts, and cited-reference relationships were analyzed. Two reviewers independently classified each publication into one of five mutually exclusive clinical decision domains. Temporal distributions were compared using Pearson's chi-square test, and a research-priority framework was synthesized by triangulating the quantitative findings with representative clinical evidence.

**Results:**

Of 778 source records, 283 publications from 1993 to 2026 were included. Reviewer agreement was 78.80% (Cohen's kappa = 0.722). Histological regression and mucosal recovery predominated in 1993–2007 (34.7%), whereas surveillance and prevention (39.6%) and risk stratification (30.2%) became dominant in 2008–2017 and remained the leading domains in 2018–2026. Domain distributions differed significantly across periods (*P* = 0.008) and remained significant after exclusion of the incomplete 2026 publication year. The principal translational gaps were the absence of externally validated multimodal risk thresholds and comparative evidence defining surveillance eligibility, intervals, duration, and outcomes.

**Conclusion:**

The field has shifted from asking whether gastric mucosal damage can recover to determining who remains at risk and how that risk should be managed. The resulting clinical decision taxonomy and research-priority framework support future validation of multimodal risk thresholds and comparative surveillance strategies; they do not establish universal surveillance or specific intervals.

## 1 Introduction

*Helicobacter pylori* infection is a major modifiable cause of chronic atrophic gastritis and gastric adenocarcinoma. Persistent infection can drive progressive glandular atrophy, intestinal metaplasia, dysplasia, and malignant transformation. Accordingly, eradication has become central to gastric cancer prevention. The Kyoto consensus recommended treatment before preneoplastic changes develop whenever feasible, while the Taipei global consensus concluded that eradication reduces gastric cancer risk and supported screen-and-treat strategies in populations and individuals at increased risk (1, 2). In patients previously treated for early gastric neoplasia, randomized evidence further showed that eradication reduced metachronous gastric cancer and improved corpus atrophy (3).

Eradication, however, does not uniformly reverse established mucosal damage or abolish subsequent cancer risk. Prospective studies have documented improvement in gastric atrophy and intestinal metaplasia after successful eradication, although the magnitude of recovery varies across gastric sites and baseline disease stages, and endoscopic improvement may occur later than histological improvement (4–6). Long-term follow-up of an eradication trial also linked cumulative persistence of infection to progression of precancerous lesions and identified incomplete intestinal metaplasia as a marker of higher malignant risk (7). Conversely, gastric cancer has been observed more than a decade after successful eradication, including during the second decade of follow-up (8, 9). Histological improvement and oncological safety therefore represent related but non-equivalent outcomes.

This residual risk has shifted clinical attention from whether gastric mucosa can recover to which patients remain vulnerable and how they should be monitored. Advanced Operative Link on Gastritis Assessment stages have been associated with subsequent gastric neoplasia, supporting structured histological risk stratification (10). Contemporary guidance increasingly combines high-quality endoscopy, systematic mucosal staging, and risk-adapted surveillance. MAPS II recommended surveillance for advanced atrophy and selected patients with intestinal metaplasia and additional risk factors, while MAPS III extended this framework using updated endoscopic and management strategies (11, 12). Recent United States guidance similarly emphasizes individualized assessment based on endoscopic, histological, demographic, and familial risk factors rather than universal surveillance (13, 14). Emerging molecular evidence suggests that gastric mucosal DNA methylation may further refine post-eradication risk stratification (15).

Previous bibliometric studies mapped clinical *Helicobacter pylori* eradication research and broad *H. pylori* research hotspots, but they relied on single-database records and conventional keyword or citation structures (16, 17). They did not isolate the post-eradication chronic atrophic gastritis setting, reconcile cited references across databases, or classify every publication according to its principal clinical decision purpose.

Despite these advances, the evidence remains distributed across studies of histological regression, residual gastric cancer, biomarkers, endoscopic surveillance, and prevention guidelines. Keyword-based mapping alone may not adequately distinguish these clinically consequential decision domains or quantify their changing prominence. We therefore conducted a multi-database bibliometric and clinical decision-domain analysis integrating Web of Science, Scopus, and PubMed with deterministic deduplication, cross-database reference normalization, network and temporal analyses, and independent article-level classification. Specifically, we aimed to: (1) characterize the growth, conceptual structure, and intellectual foundations of the field; (2) quantify the temporal transition among clinically relevant decision domains, particularly from mucosal recovery toward residual-risk assessment and surveillance; and (3) synthesize the convergent findings into a clinically oriented, hypothesis-generating framework of unresolved decisions and priority study designs.

## 2 Materials and methods

### 2.1 Data sources, search strategy, and eligibility

This study was designed as a systematic multi-database bibliometric/scientometric analysis with independent article-level clinical decision-domain classification. Web of Science Core Collection, Scopus, and PubMed were searched on 27 July 2026. The strategy combined three concept domains: chronic atrophic gastritis or gastric atrophy; *Helicobacter pylori* eradication or the post-eradication setting; and clinically relevant outcomes including histological regression, mucosal recovery, gastric precancerous lesions, residual gastric cancer risk, risk stratification, surveillance, OLGA/OLGIM staging, and pepsinogen-related biomarkers. Database-specific field tags and controlled vocabulary were applied. Web of Science was restricted to articles and reviews indexed in the Science Citation Index Expanded, Scopus to journal articles and reviews, and PubMed searches combined Medical Subject Headings with title/abstract terms. No year or language restriction was applied at retrieval or screening, and language was not used as an exclusion criterion. Complete search strategies are provided in Supplementary Table S1.

Eligible publications were required to substantively address all three domains. Studies of gastric cancer without a substantive *H. pylori* eradication or post-eradication context were not eligible. Records limited to eradication regimens, antimicrobial resistance, gastric cancer treatment, or unrelated outcomes were excluded.

### 2.2 Deduplication, screening, and metadata standardization

Source-native identifiers were preserved, including Web of Science accession numbers, Scopus EIDs, and PubMed PMIDs. Cross-database duplicates were resolved deterministically using normalized DOI, PMID, and exact normalized title. Fuzzy title similarity only flagged records for manual review and was never used for automatic deletion.

An artificial intelligence-assisted high-sensitivity prescreen was used solely to prioritize the order of manual review and did not exclude or remove any record. All 420 deduplicated bibliographic entities underwent independent title-and-abstract assessment by both reviewers; disagreements and uncertain records were resolved by consensus, and full texts were consulted when bibliographic information was insufficient.

Metadata were reconstructed from the original exports and mapped to locked entity identifiers. Original fields remained unchanged; reviewed derivative fields were created for authors, affiliations, institutions, countries/regions, sources, and keywords. Only unambiguous variants were merged, and country/region data were derived exclusively from verified affiliations. Database-generated slash-form indexing expressions were removed; 252 publications ultimately contributed 571 unique author keywords and 1,646 document-keyword associations.

### 2.3 Descriptive, collaboration, and keyword analyses

Annual and cumulative output, source distribution, authorship, and productivity were calculated using full counting. A three-year trailing moving average and compound annual growth rate were estimated for complete years through 2025; 2026 was treated as incomplete and excluded from primary trend and burst calculations.

Country/region and author collaboration networks were constructed in VOSviewer version 1.6.20 (18) using full counting and association-strength normalization, with minimum publication thresholds of three and five, respectively. Institutional collaboration was summarized with a shared-publication matrix. Author-keyword co-occurrence networks were constructed in VOSviewer from cleaned author keywords using full counting and association-strength normalization. Keywords occurring in at least five publications were retained, and the resulting network was used for network, temporal-overlay, and density visualizations.

### 2.4 Clinical decision-domain classification

To quantify clinically meaningful shifts that could not be captured reliably by keyword networks alone, all included publications were classified into one of five mutually exclusive domains: histological regression and mucosal recovery; residual gastric cancer risk; biomarkers and risk stratification; endoscopic surveillance and cancer prevention; or mixed or other clinically relevant focus.

Two reviewers independently assigned each publication according to its principal objective, primary outcome, and main conclusion. The classification was performed at the publication level and was not intended to categorize patient-level comorbidities; comorbid conditions were therefore not treated as a separate decision-domain axis. The mixed/other category comprised eligible publications with multiple co-primary domains or another eligible clinical focus not dominated by the four principal categories; representative examples included mechanistic, treatment-related, microbiome, MALT lymphoma, and mixed clinical questions. Full texts were consulted when needed. Raw agreement and Cohen's kappa (19) were calculated, and discordant classifications were resolved by consensus, with the final rationale retained in an audit file.

The same three periods used throughout the temporal analyses—1993–2007, 2008–2017, and 2018–2026—were applied to the clinical classification comparison. These intervals represented the early formation, subsequent expansion, and contemporary phases of the evidence base while maintaining interpretable publication counts, rather than equal-duration calendar windows. Because the temporal comparison evaluated within-period domain proportions rather than raw annual publication counts, simple annualization would not alter the proportional distribution. Domain distributions were compared using Pearson's chi-square test, and effect size was assessed using Cramer's V. Expected cell frequencies were examined before interpretation of the Pearson test; one of 15 cells (6.7%) had an expected count below 5 (minimum, 4.50), and none had an expected count below 1. Two-sided 95% confidence intervals for domain proportions were calculated using the Wilson score method. A sensitivity analysis excluded the incomplete 2026 publication year.

### 2.5 Reference normalization, co-citation, and bibliographic coupling

Reference-based analyses were restricted to publications with usable cited-reference data. Web of Science and Scopus references were parsed separately and reconciled hierarchically using normalized DOI, PMID, a composite key of first-author surname, year, journal, volume, and starting page, or a normalized original reference string. Duplicate references within the same citing publication were counted once.

Local citation frequency was defined as the number of included publications citing a normalized reference. The 15 most frequently locally cited references were displayed. Reference co-citation networks were constructed from normalized references cited locally by at least 15 included publications. Bibliographic coupling linked publications sharing at least five normalized references, after which the 100 connected publications with the highest total link strength were retained. Both networks were visualized in VOSviewer using full counting and association-strength normalization.

### 2.6 Thematic mapping, keyword timeline, and burst detection

Thematic structure was evaluated using the thematicMap() function in Bibliometrix version 5.4.1 (20). Louvain clustering (21), full counting, unigram terms, no stemming, and a fixed seed of 1234 were used. Candidate minimum-frequency thresholds of 3, 5, 7, and 10 were compared, and the final solution was selected according to thematic interpretability and network parsimony. Themes were positioned by Callon centrality and density as motor, basic, niche, or emerging/declining themes.

The CiteSpace keyword co-occurrence network was constructed in CiteSpace version 7.0.1 (22) using one-year time slices from 1993 to 2026, cosine similarity, g-index node selection with k = 25, and no pruning. Cluster labels were generated using the log-likelihood ratio algorithm and refined from ranked cluster terms when necessary. Modularity Q and weighted mean silhouette were used to assess cluster quality.

Keyword and cited-reference bursts were detected in R using a Kleinberg-style multistate Poisson model (23). Primary analyses covered 1993–2025, with s = 2, gamma = 1, and a minimum duration of two years. Keywords occurring in at least three publications and references cited by at least five publications were eligible. For entities with multiple intervals, the strongest burst was retained.

### 2.7 Clinical research-priority synthesis

After completion of the quantitative analyses, the authors triangulated the article-level domain distribution, keyword timeline, burst patterns, geographical structure, and representative clinical studies and guidelines. For each principal decision area, we summarized what the literature supported, the unresolved clinical decision, and the study design most directly capable of addressing it. This synthesis was not an evidence-grade assessment and was intended for hypothesis generation and research planning.

### 2.8 Software, statistical analysis, reproducibility, and artificial intelligence-assisted tools

Data processing, statistical testing, and most visualizations were conducted in R version 4.6.1; Bibliometrix version 5.4.1, CiteSpace version 7.0.1, and VOSviewer version 1.6.20 were used as specified above. Tests were two-sided with *P* < 0.05. Because the study was designed as a bibliometric/scientometric analysis rather than an effect-estimating systematic review or meta-analysis, no prospective review protocol was registered. Original exports, locked identities, screening and consensus records, terminology dictionaries, crosswalks, scripts, and checksum manifests were preserved for auditability and reproducibility. OpenAI ChatGPT (web application; GPT-5-series models; OpenAI, San Francisco, CA, United States; accessed July–August 2026) was used to assist with high-sensitivity prescreen prioritization, drafting R code, language editing, and manuscript organization. It was not used to make final eligibility decisions, assign clinical decision domains, or determine final analyses or interpretations. All code, outputs, citations, and text were checked against the source data and approved by the authors, who take full responsibility for the work. Because this study analyzed published bibliographic records and did not involve human participants or animals, ethics approval and informed consent were not required.

## 3 Results

### 3.1 Study selection and corpus characteristics

The searches retrieved 343 records from Web of Science, 278 from Scopus, and 208 from PubMed. After source and document-type restrictions, 778 records were exported and deterministically consolidated into 420 unique entities.

Following independent screening and consensus, 283 publications were included and 137 excluded. The main reasons were absence of a substantive *Helicobacter pylori* eradication context (*n* = 77), no chronic atrophic gastritis or gastric atrophy focus (*n* = 31), no eligible outcome (*n* = 15), or restriction to eradication regimens or antimicrobial resistance (*n* = 9); five further records concerned gastric cancer treatment or ineligible document types (Figure 1A; Supplementary Table S2).

**Figure 1:**
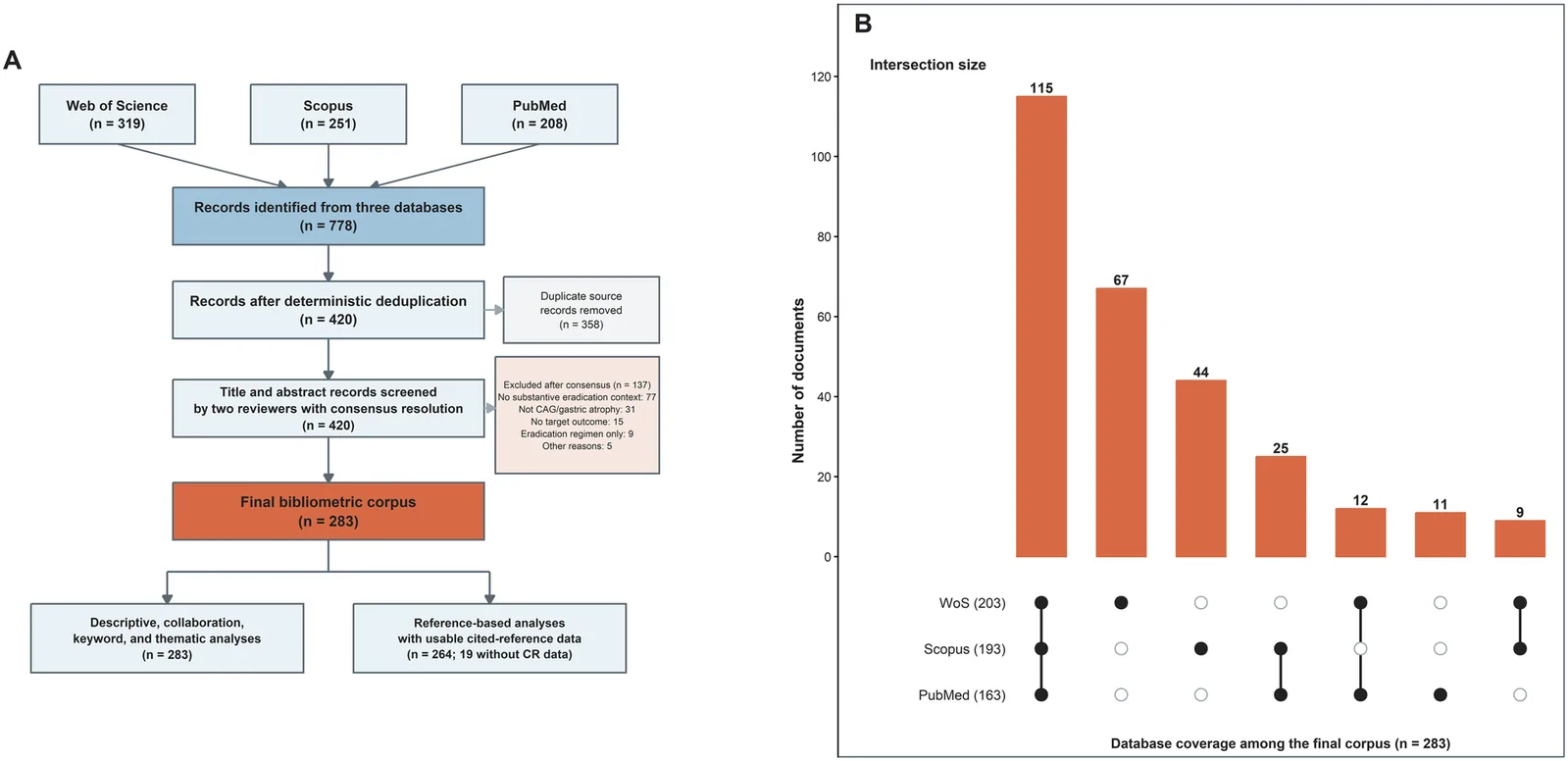
Study selection nnnnand database coverage. **(A)** Flow diagram of multi-database retrieval, deterministic deduplication, and eligibility screening. The database searches yielded 343 records from Web of Science, 278 from Scopus, and 208 from PubMed. After database-specific restrictions, 778 source records were exported and consolidated into 420 unique bibliographic entities. Following independent title-and-abstract screening and consensus resolution, 283 publications were included. **(B)** UpSet plot showing the coverage and intersections of the 283 included publications across Web of Science, Scopus, and PubMed. Vertical bars represent the number of publications in each database intersection, and horizontal bars represent the total number indexed by each database.

Among included publications, 203 were indexed in Web of Science, 193 in Scopus, and 163 in PubMed; only 115 occurred in all three databases, whereas 122 were retrieved from a single database (Figure 1B). The corpus spanned 1993–2026, included 1,456 authors across 136 sources, and had usable cited-reference data for 264 publications. International collaboration occurred in 57 publications (20.14%). Detailed geographical, author, and institutional patterns are shown in Supplementary Figures S1-S2.

### 3.2 Publication growth and transition in clinical decision domains

Annual publication output was low during the early study period, increased progressively after 2000, and showed more sustained growth after approximately 2010. The three-year trailing moving average confirmed a long-term upward trajectory despite annual fluctuations (Figure 2A). Based on complete calendar years from 1993 through 2025, the compound annual growth rate was 9.45% (Figure 2B). Nineteen publications from the incomplete 2026 calendar year were displayed separately and excluded from primary complete-year trend calculations.

**Figure 2:**
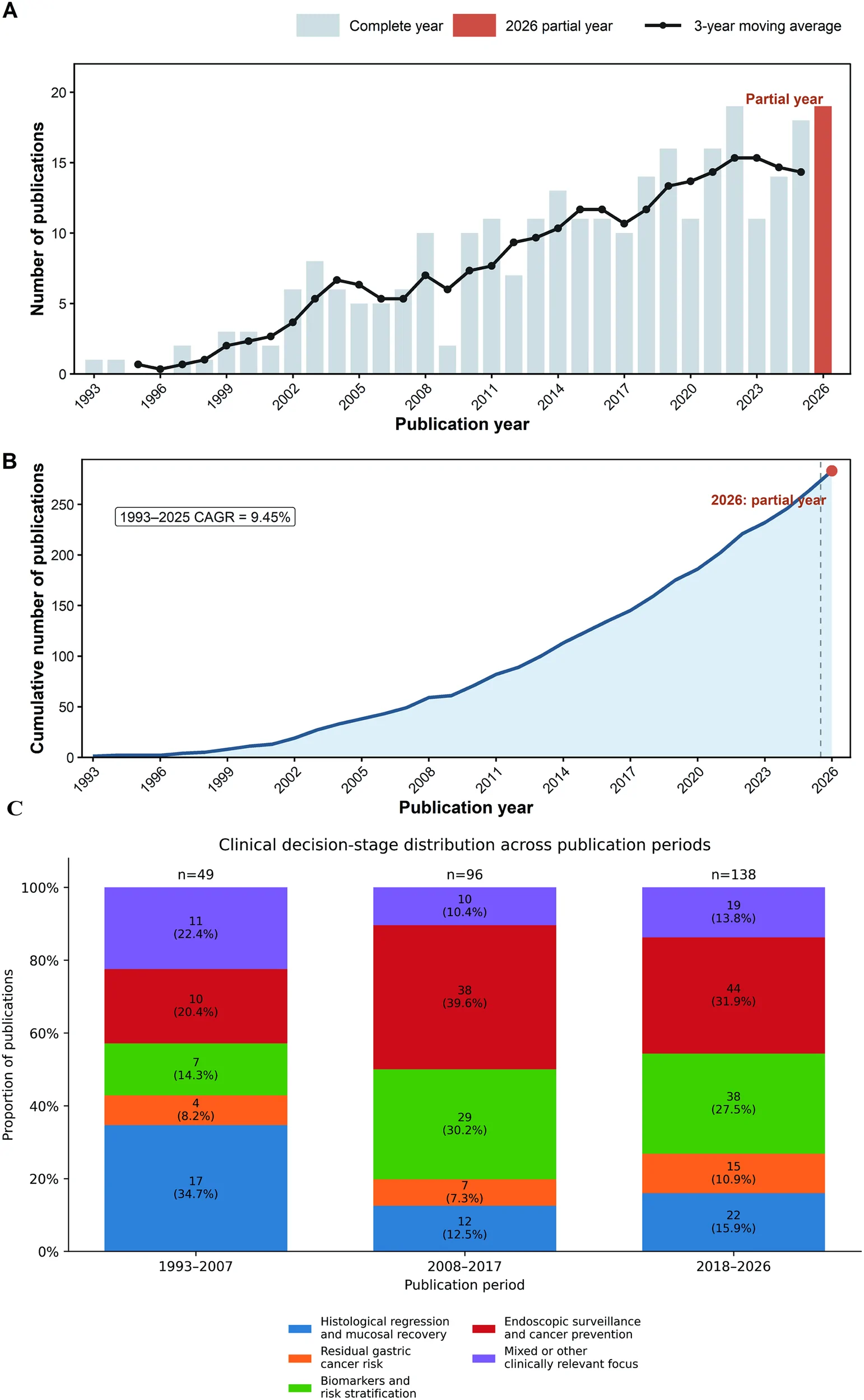
Temporal evolution of publication output and clinical decision domains in research on chronic atrophic gastritis after *Helicobacter pylori* eradication, 1993-2026. **(A)** Annual publication output and the three-year trailing moving average. Publications from 2026 are highlighted separately because the year was incomplete at the time of retrieval and were excluded from the moving-average calculation. **(B)** Cumulative publication output over the study period. The compound annual growth rate was calculated using complete calendar years from 1993 through 2025. **(C)** Distribution of the five independently classified clinical decision domains across 1993−2007, 2008−2017, and 2018−2026. The respective period totals were 49, 96, and 138 publications. Counts and percentages are displayed within the stacked bars. Domain distributions differed significantly across periods (Pearson's chi-square = 20.764, df = 8, *P* = 0.008; Cramer's V = 0.192). The association remained significant after exclusion of the incomplete 2026 publication year (chi-square = 22.056, df = 8, *P* = 0.005).

The clinical decision-domain analysis showed a distinct redistribution of research priorities over time. Endoscopic surveillance and cancer prevention was the largest domain overall, comprising 92 publications (32.5%). Biomarkers and risk stratification accounted for 74 publications (26.1%), histological regression and mucosal recovery for 51 (18.0%), mixed or other clinically relevant topics for 40 (14.1%), and residual gastric cancer risk for 26 (9.2%) (Table 2; Supplementary Table S6).

The reviewers independently assigned the same domain to 223 of 283 publications, corresponding to 78.80% raw agreement and a Cohen's kappa of 0.722. All 60 discordant records were resolved by consensus.

The distribution of clinical decision domains differed significantly across the three periods (chi-square = 20.764, df = 8, *P* = 0.008; Cramer's V = 0.192) (Figure 2C). The Pearson assumptions were considered acceptable: only one of 15 expected cell counts was below 5 (minimum, 4.50), and none was below 1. Histological regression and mucosal recovery predominated during 1993–2007, accounting for 34.7% of publications. During 2008–2017, its proportion declined to 12.5%, whereas endoscopic surveillance and cancer prevention and biomarkers and risk stratification increased to 39.6% and 30.2%, respectively. These two domains remained predominant during 2018–2026, accounting for 31.9% and 27.5%, while residual gastric cancer risk reached 10.9%. Two-sided 95% Wilson score confidence intervals for the overall and period-specific domain proportions are provided in Supplementary Table S6.

The association remained significant after exclusion of the incomplete 2026 publication year (chi-square = 22.056, df = 8, *P* = 0.005). These findings provide article-level quantitative evidence of a progressive redistribution from histological recovery toward post-eradication risk assessment, surveillance, and gastric cancer prevention.

### 3.3 Conceptual structure and temporal development

After standardization and removal of slash-form indexing expressions, 252 publications contributed 571 unique author keywords and 1,646 document-keyword associations. Forty-four keywords occurring at least five times formed a network with 466 links and total link strength of 1,918 (Figure 3A).

**Figure 3:**
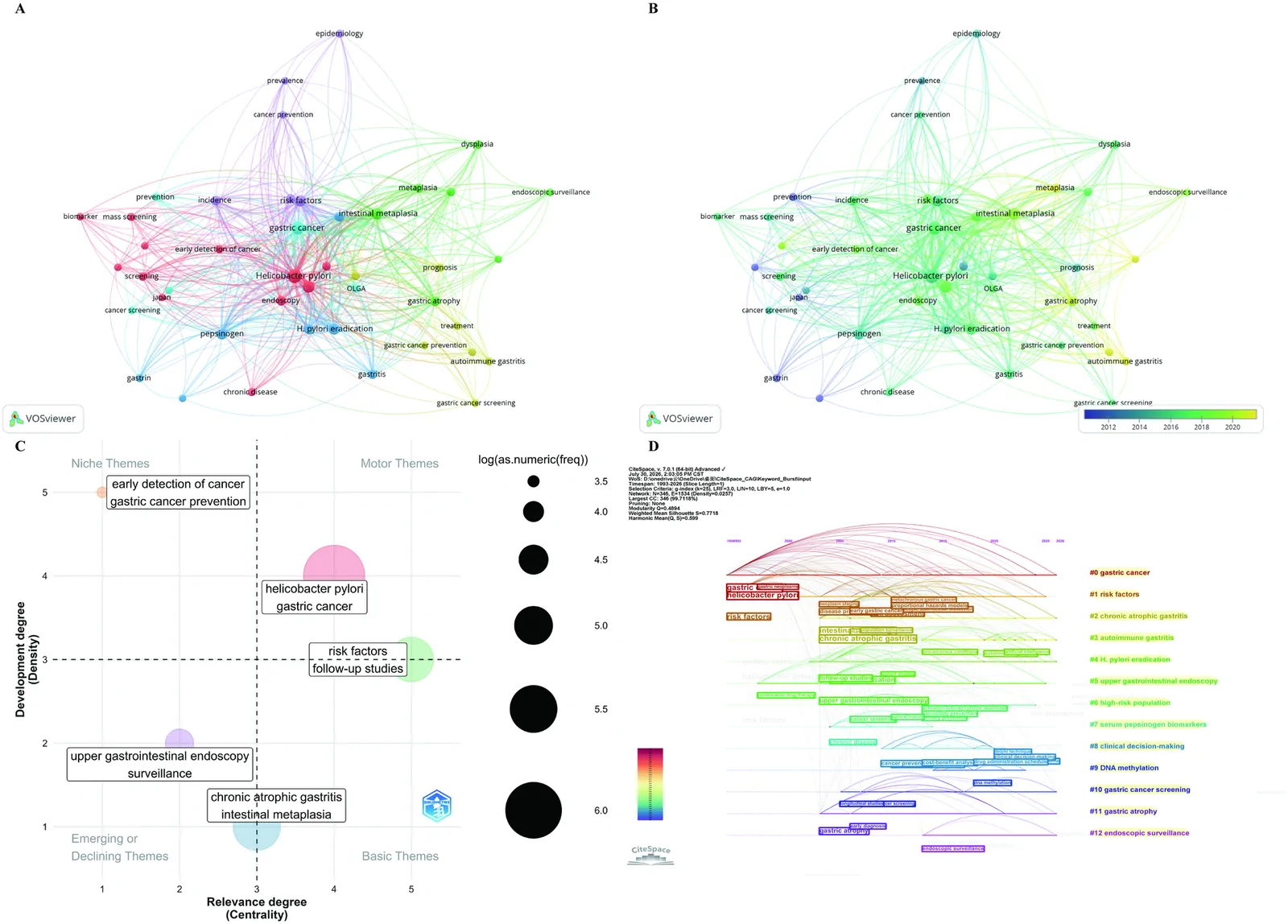
Conceptual structure and temporal evolution of research on chronic atrophic gastritis after *Helicobacter pylori* eradication. **(A)** Author-keyword co-occurrence network. Cleaned author keywords occurring in at least five publications were included. The connected network comprised 44 keywords, 466 links, and a total link strength of 1,918. Node size represents occurrence frequency, line thickness represents co-occurrence strength, and node color represents the cluster identified by VOSviewer. **(B)** Temporal overlay visualization of the author-keyword network. Node color represents the average publication year of each keyword. The displayed color range was restricted to 2011−2021 to improve discrimination of temporal variation. **(C)** Thematic strategic map based on cleaned author keywords. Themes are positioned according to Callon centrality and Callon density. The upper-right, lower-right, upper-left, and lower-left quadrants represent motor, basic, niche, and emerging or declining themes, respectively. Bubble size reflects theme frequency, and labels indicate representative keywords. Placement in the emerging/declining quadrant reflects relative keyword centrality and density and should not be interpreted as evidence of declining clinical importance. **(D)** CiteSpace keyword-cluster timeline showing the longitudinal development of major themes from 1993 to 2026. The network contained 346 nodes, 1,534 links, and 24 clusters; the 13 largest interpretable clusters are displayed. Horizontal lines represent keyword clusters, while nodes and curved links indicate keyword occurrence and interconnections across annual time slices. Cluster labels were generated primarily using the log-likelihood ratio algorithm and refined from ranked cluster terms when necessary. The modularity Q and weighted mean silhouette values were 0.4894 and 0.7718, respectively.

The network centered on *Helicobacter pylori*, gastric cancer, chronic atrophic gastritis, *H. pylori* eradication, intestinal metaplasia, gastric atrophy, pepsinogen, risk factors, screening, and endoscopic surveillance. Earlier average publication years characterized pepsinogen, gastrin, epidemiology, and treatment terms, whereas precancerous conditions, prognosis, risk factors, surveillance, and cancer prevention were more recent (Figure 3B).

The strategic map identified *H. pylori* and gastric cancer as a motor theme; chronic atrophic gastritis and intestinal metaplasia as basic themes; and endoscopy and surveillance as an emerging-or-declining theme with limited consolidation rather than necessarily declining clinical importance (Figure 3C).

The CiteSpace timeline contained 346 nodes, 1,534 links, and 24 clusters (modularity Q = 0.4894; weighted mean silhouette = 0.7718); the 13 largest interpretable clusters were displayed (Figure 3D). Earlier clusters emphasized pepsinogen, gastric cancer, and high-risk populations, whereas later clusters incorporated eradication, clinical decision-making, DNA methylation, gastric atrophy, screening, and surveillance.

### 3.4 Intellectual foundations and contemporary research fronts

Reference normalization across 264 publications produced 18,188 document-reference associations and 10,110 unique reference identities.

Uemura et al. (24) was the most locally cited publication (104 citations), followed by Dixon et al. (25) (88) and Wong et al. (26) (70). Other leading works concerned gastric carcinogenesis, histopathology, eradication outcomes, premalignant lesions, and surveillance guidance (Figure 4A).

**Figure 4:**
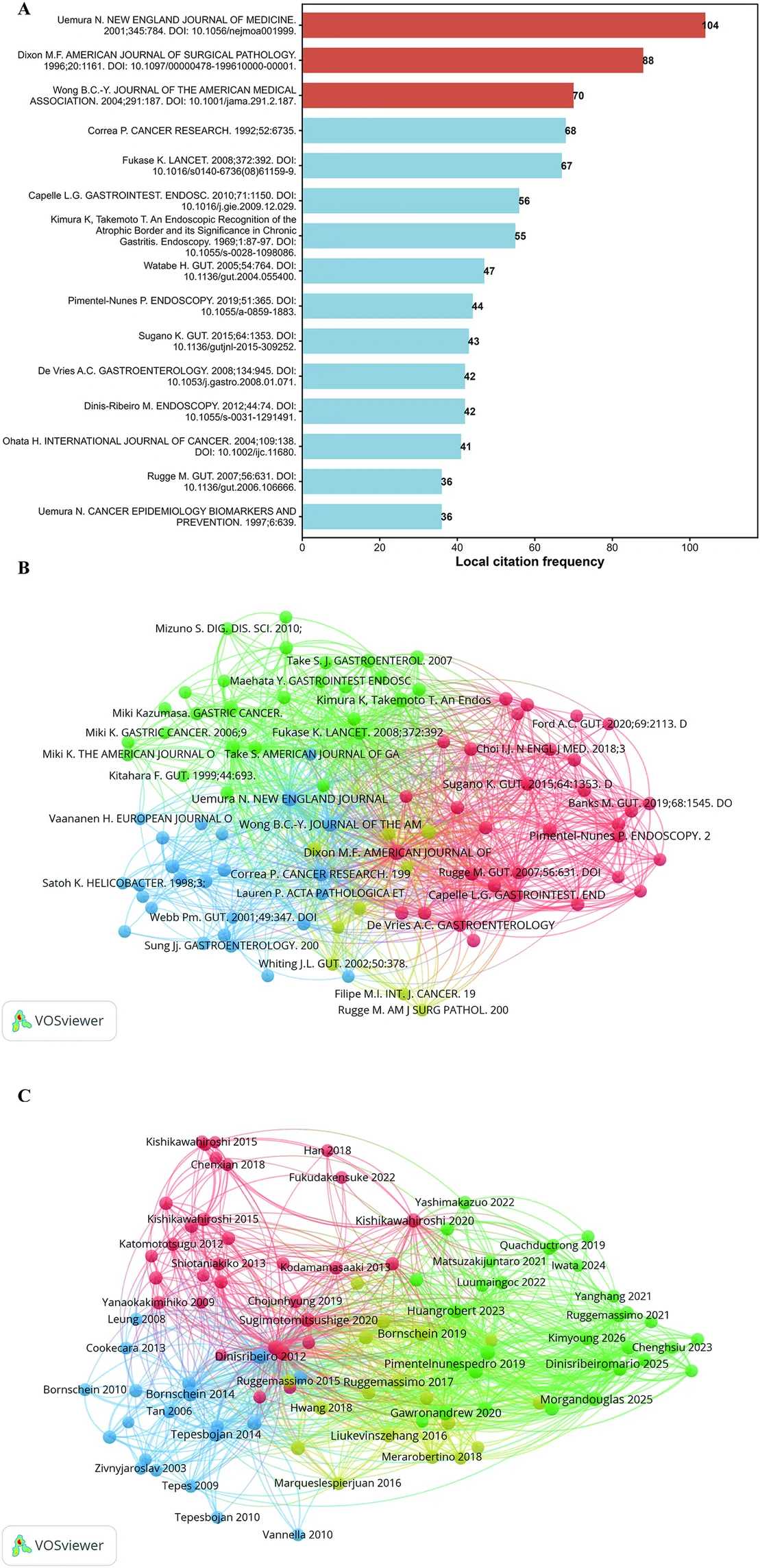
Intellectual foundations and bibliographic-coupling structure of research on chronic atrophic gastritis after helicobacter pylori eradication. **(A)** The 15 references with the highest local citation frequencies. Local citation frequency was defined as the number of publications within the final corpus citing a normalized reference. The three most frequently locally cited references are highlighted. **(B)** Reference co-citation network. References with at least 15 local citations were included, resulting in 93 nodes, four clusters, 3,703 links, and a total link strength of 16,253. Node size represents local citation frequency, line thickness represents co-citation strength, and node color represents the cluster identified by VOSviewer. **(C)** Bibliographic-coupling network of the 100 connected publications with the highest total link strength, selected from the network requiring at least five shared references. The final network comprised four clusters, 2,207 links, and a total edge weight of 18,628. Node size represents total link strength, line thickness represents the number of shared references, and node color represents the VOSviewer cluster.

The co-citation network comprised 93 references, four clusters, 3,703 links, and total link strength of 16,253 (Figure 4B). Bibliographic coupling retained the 100 publications with highest total link strength from 237 connected records, yielding four clusters, 2,207 links, and total edge weight of 18,628 (Figure 4C).

### 3.5 Burst patterns and emerging priorities

Thirteen author keywords met burst criteria (Supplementary Figure S4A; Supplementary Table S5). Gastric cancer showed the strongest burst (18.20; 2013−2025), followed by *Helicobacter pylori* (15.42; 2016−2025), intestinal metaplasia (11.49; 2013−2025), and chronic atrophic gastritis (10.03; 2020−2025); nine bursts remained active through 2025.

The strongest cited-reference bursts were concentrated in recent guidelines, consensus statements, and evidence syntheses addressing premalignant conditions, eradication, gastric cancer risk, and surveillance (Supplementary Figure S4B; Supplementary Table S5). These recent keyword and cited-reference bursts converged with the article-level clinical classification: the persistence of gastric cancer, intestinal metaplasia, and guideline-related research fronts paralleled the increasing prominence of biomarkers and risk stratification and endoscopic surveillance and cancer prevention. This convergence supports a common temporal interpretation across complementary analytical approaches rather than implying a causal relationship between bibliometric indicators and clinical outcomes.

### 3.6 Clinical evidence gaps and research priorities

Triangulation of the clinical classification, thematic analyses, burst patterns, and geographical structure showed that the principal translational deficit was not a lack of candidate markers or surveillance statements, but limited evidence linking risk estimates to externally validated clinical decisions. Five recurring gaps were identified (Table 1): whether histological regression predicts durable cancer-risk reduction; how absolute risk varies by baseline phenotype and time since eradication; which multimodal models remain calibrated across populations; which patients benefit from specific surveillance strategies; and whether current pathways are transferable beyond high-incidence settings.

**Table 1:** Clinically oriented evidence gaps and priority research designs after *Helicobacter pylori* eradication.

Clinical decision area	What current evidence supports	Decision-critical gap	Priority research design
Histological recovery	Partial regression of atrophy and intestinal metaplasia can occur after eradication, but recovery is heterogeneous across lesions, sites, and follow-up intervals (3–7).	Whether mucosal regression predicts a durable reduction in gastric cancer risk, and which pathological changes remain reversible.	Prospective cohorts with standardized serial endoscopy, mapping biopsies, central pathology review, and long-term cancer outcomes.
Residual gastric cancer risk	Gastric cancer can occur more than a decade after eradication, and metachronous risk persists after treatment of early gastric neoplasia (8, 9, 28).	Absolute risk according to baseline mucosal phenotype, previous neoplasia, and time elapsed since eradication.	Large multinational long-term cohorts using harmonized definitions, competing-risk methods, and clearly separated primary, recurrent, and metachronous outcomes.
Risk stratification	Histological staging, pepsinogen, endoscopic phenotypes, and gastric mucosal methylation show prognostic potential (10, 15, 29–32).	Which multimodal combination provides externally valid, calibrated, and clinically actionable risk thresholds across populations.	External validation and recalibration of prespecified multimodal models, followed by decision-curve and clinical-impact analyses.
Endoscopic surveillance	Guidelines support risk-adapted follow-up for selected patients with advanced premalignant changes or other high-risk features (1, 2, 11–14).	Eligibility, interval, duration, stopping rules, imaging and biopsy protocols, and the balance of benefit, burden, and cost.	Prospective comparative studies or pragmatic trials reporting stage at detection, curative treatment, mortality, adherence, procedural burden, harms, and cost-effectiveness.
Generalizability and implementation	The evidence base is concentrated in East Asia and Europe, while international collaboration remains limited.	Transportability of risk thresholds and surveillance pathways to lower-incidence, ethnically diverse, and resource-constrained settings.	Multinational implementation studies using common data elements, standardized outcomes, and prespecified assessment of regional and population heterogeneity.

This table is an interpretive synthesis generated by triangulating the article-level clinical classification, thematic and burst analyses, geographical patterns, and representative clinical evidence. It is intended to support hypothesis generation and research planning, not to provide evidence-graded recommendations or specific surveillance intervals.

**Table 2:** Distribution of clinical decision domains overall and across publication periods.

Clinical decision domain	Primary scope	Overall, *n* (%)	1993−2007, *n* (%)	2008–2017, *n* (%)	2018–2026, *n* (%)
Histological regression and mucosal recovery	Changes in gastric atrophy, intestinal metaplasia, histological scores, or mucosal recovery after eradication	51 (18.0)	17 (34.7)	12 (12.5)	22 (15.9)
Residual gastric cancer risk	Incident, *de novo*, recurrent, or metachronous gastric cancer after eradication	26 (9.2)	4 (8.2)	7 (7.3)	15 (10.9)
Biomarkers and risk stratification	Histological staging, serological, endoscopic, molecular, or multivariable risk assessment	74 (26.1)	7 (14.3)	29 (30.2)	38 (27.5)
Endoscopic surveillance and cancer prevention	Endoscopic screening, surveillance, guideline implementation, early detection, or cancer-prevention strategies	92 (32.5)	10 (20.4)	38 (39.6)	44 (31.9)
Mixed or other clinically relevant focus	Multiple co-primary domains or other eligible clinical questions not dominated by the four categories above	40 (14.1)	11 (22.4)	10 (10.4)	19 (13.8)
Total	**-**	**283** (**100)**	**49** (**100)**	**96** (**100)**	**138** (**100)**

Each publication was assigned to one mutually exclusive domain according to its principal objective, primary outcome, and main conclusion. Two reviewers independently classified all 283 publications. Raw agreement was 78.80%, and Cohen's kappa was 0.722. The 60 discordant classifications were resolved by consensus. Domain distributions differed across periods (Pearson's chi-square = 20.764, df = 8, *P* = 0.008; Cramer's V = 0.192). Exclusion of publications from the incomplete 2026 calendar year yielded consistent results (chi-square = 22.056, df = 8, *P* = 0.005). Two-sided 95% Wilson score confidence intervals for the domain proportions are provided in Supplementary Table S6.

The corresponding priorities were standardized longitudinal cohorts with cancer outcomes, multinational validation and impact assessment of multimodal models, prospective comparative surveillance studies, and implementation and cost-effectiveness research. These priorities are hypothesis-generating rather than evidence-graded recommendations.

## 4 Discussion

### 4.1 Principal findings

This multi-database bibliometric and clinical decision-domain analysis contributes two outputs beyond conventional science mapping: a reproducible five-domain clinical decision taxonomy and a research-priority framework linking the observed transition to unresolved clinical decisions. Research on chronic atrophic gastritis after *Helicobacter pylori* eradication progressively moved from evaluating mucosal reversibility toward managing persistent gastric cancer risk. Histological regression and mucosal recovery predominated during 1993–2007, whereas biomarkers and risk stratification and endoscopic surveillance and cancer prevention became the leading domains thereafter. The significant temporal difference, together with a modest Cramer's V, indicates a gradual redistribution of priorities rather than abrupt replacement of one agenda by another.

The bibliometric findings supported the same interpretation. Earlier research was anchored in gastric carcinogenesis, pathological assessment, serum pepsinogen, and eradication outcomes. More recent keyword clusters and citation bursts increasingly involved intestinal metaplasia, residual cancer risk, risk assessment, endoscopic surveillance, and guideline-directed prevention. The central question has therefore evolved from whether eradication can improve gastric mucosal damage to which patients remain at risk and how that risk should be evaluated and monitored. This bibliometric transition should not be interpreted as a patient-level quantitative correlation among eradication, histological change, and cancer incidence; rather, it reflects a shift from assessing mucosal reversibility toward determining whether residual structural and molecular abnormalities continue to confer clinically meaningful cancer risk after eradication.

### 4.2 From histological recovery to residual gastric cancer risk

The early emphasis on histological regression reflected uncertainty regarding reversibility of the gastric carcinogenic cascade. Removing *H. pylori* may improve inflammation, glandular atrophy, and, in selected patients, intestinal metaplasia, although recovery varies by gastric location, baseline severity, and endpoint (5, 6). Long-term trials also linked persistent infection to progression of precancerous lesions and successful eradication to more favorable mucosal outcomes (7, 27). In patients treated endoscopically for early gastric neoplasia, eradication reduced metachronous cancer and improved corpus atrophy (3).

Histological improvement, however, is not equivalent to elimination of cancer risk. Post-eradication mucosal evolution is heterogeneous rather than uniformly reversible. Gastric atrophy and intestinal metaplasia may partially regress after successful eradication, but the extent of recovery varies according to baseline severity, anatomical site, and follow-up duration (5–7, 27). Conversely, persistent or advanced intestinal metaplasia may continue to mark a gastric mucosal field at increased neoplastic risk. Advanced atrophy, established intestinal metaplasia, clonal alterations, and epigenetic field defects may persist after bacterial clearance. Gastric cancer has occurred more than a decade after eradication (8, 9), and substantial metachronous risk persisted after endoscopic resection despite successful eradication, particularly in men and patients with severe atrophy or multiple previous cancers (28). Histological improvement should therefore be regarded as a favorable mucosal outcome rather than a validated surrogate for elimination of future gastric cancer risk.

Residual gastric cancer risk represented a smaller share of the corpus than surveillance or biomarker research, but long-term incidence studies require large cohorts, verified eradication status, prolonged follow-up, and precise outcome definitions. Limited publication volume may therefore reflect methodological difficulty rather than low clinical importance; persistent risk is the rationale for risk stratification and surveillance. Whether specific degrees of mucosal regression translate into durable reductions in cancer incidence remains unresolved, corresponding to the histological recovery and residual gastric cancer risk priorities summarized in Table 1.

### 4.3 Risk stratification and surveillance after eradication

Post-eradication risk is heterogeneous. Advanced Operative Link on Gastritis Assessment stages have been associated with gastric neoplastic progression (10), and prospective evidence supports gastritis staging in secondary prevention (29). Histology remains imperfect because atrophy and intestinal metaplasia may be patchy and depend on biopsy quality and interpretation.

Endoscopic phenotypes and biomarkers may add complementary information. Serum pepsinogen has predicted metachronous gastric cancer after endoscopic treatment (30). In a population-based post-eradication programme, a two-stage serology-endoscopy strategy showed that gastric mucosal DNA methylation discriminated cancer or extensive premalignant lesions better than pepsinogen alone (31). Persistent methylation abnormalities have been investigated as markers of field carcinogenesis (32), and prospective evidence suggests particular value among eradicated individuals with open-type atrophy (15).

Future models will probably need to combine OLGA or OLGIM stage, endoscopic extent of atrophy or intestinal metaplasia, age, sex, family history, smoking, previous neoplasia, serological markers, and molecular-field alterations. Primary-prevention populations should be distinguished from patients previously treated for gastric neoplasia because baseline risks and surveillance thresholds differ.

Endoscopic surveillance and cancer prevention constituted the largest clinical domain, consistent with strong guideline and consensus bursts. Kyoto and Taipei strengthened eradication-based prevention while recognizing continued risk assessment in patients with established damage (1, 2). MAPS II and III refined surveillance according to premalignant extent, additional risk factors, and high-quality endoscopy (11, 12), while recent United States guidance emphasized individualized rather than universal surveillance (13, 14).

The emerging-or-declining position of surveillance in the strategic map should not be read as declining clinical importance. Strategic maps reflect keyword centrality and cohesion, not evidence quality. Together with manual classification, the timeline, and citation bursts, the result more plausibly indicates an important but incompletely consolidated field with uncertainty regarding eligibility, intervals, biopsy protocols, image-enhanced endoscopy, and stopping rules. Accordingly, the risk-stratification and surveillance priorities in Table 1 emphasize external validation of multimodal models and prospective comparison of risk-adapted surveillance strategies.

### 4.4 Geographic patterns and future evidence needs

East Asian countries, particularly Japan, contributed substantially to post-eradication cancer-risk and endoscopic research, while European groups shaped histological staging and surveillance guidance and North American work increasingly addressed targeted prevention in heterogeneous populations. Only 20.14% of publications involved international collaboration, potentially limiting transportability of risk thresholds across settings. This concern corresponds to the generalizability and implementation priority summarized in Table 1.

The integrated evidence map identified five decision-critical gaps and corresponding research designs (Table 1). Across domains, the central translational problem was the absence of externally transportable thresholds that connect histological, endoscopic, serological, or molecular risk estimates to specific management actions. Immediate priorities include harmonized multinational cohorts with standardized phenotyping and cancer outcomes, external validation and clinical-impact testing of multimodal models, and prospective comparison of risk-adapted surveillance strategies.

Surveillance studies should report clinically meaningful endpoints, including stage at detection, curative treatment, gastric cancer mortality, procedural burden, adherence, harms, and cost-effectiveness. The goal is not surveillance of every eradicated patient, but identification of thresholds at which expected benefit outweighs burden and remains feasible across health systems.

### 4.5 Strengths and limitations

Strengths include integration of three databases, deterministic entity resolution, cross-database reference normalization, and independent article-level classification. Raw agreement of 78.80% and Cohen's kappa of 0.722 supported reproducibility, and consensus adjudication completed all classifications. The clinically oriented synthesis further made the translational implications explicit by linking each decision domain to a specific unresolved question and study design.

Limitations include dependence on database coverage and metadata quality, preferential indexing of English-language and internationally visible literature, and an incomplete 2026 publication year. The three analytical periods were unequal in duration and were selected for historical interpretability rather than equal calendar length; this may affect the precision of period-specific estimates, although it does not alter the within-period proportions used in the primary comparison. Keyword and reference analyses excluded records lacking usable data, and assigning one principal clinical domain simplified multi-objective studies. Citation and network indicators measure visibility rather than methodological quality or clinical effectiveness. The research-priority framework is an interpretive, hypothesis-generating synthesis rather than a formal evidence-grade or stakeholder-consensus exercise; this study did not assess risk of bias or estimate intervention effects through meta-analysis.

## 5 Conclusion

Research on chronic atrophic gastritis after *Helicobacter pylori* eradication has progressively shifted from evaluating mucosal recovery toward understanding and managing residual gastric cancer risk. The principal translational priorities are externally validated multimodal risk thresholds, standardized phenotyping, comparative evidence for risk-adapted surveillance, and multinational implementation studies. Successful eradication should not be assumed to eliminate subsequent cancer risk in patients with established atrophy, intestinal metaplasia, previous gastric neoplasia, or other high-risk features; however, the present findings do not support universal surveillance or any specific surveillance interval.

## Data Availability

The data analyzed in this study is subject to the following licenses/restrictions: Owing to database licensing restrictions, the original bibliographic exports from Web of Science Core Collection and Scopus cannot be redistributed publicly. Derived datasets, row-level classification audit records, and analytical code supporting the reported findings are available from the corresponding author upon reasonable request, subject to applicable database-license conditions. Requests to access these datasets should be directed to LH, Affiliated Hospital of Jiangxi University of Chinese Medicine. heling118@126.com. Requests for access to the derived datasets, audit records, and analytical code may be directed to the corresponding author.

## Supplementary material

Supplementary Figure S1 Global publication distribution, single-country and multiple-country publication composition, and country/region collaboration network.

Supplementary Figure S2 Productive authors, author collaboration, productive institutions, and institutional collaboration matrix.

Supplementary Figure S3 Author-keyword density visualization.

Supplementary Figure S4 Keyword and cited-reference bursts in research on chronic atrophic gastritis after *Helicobacter pylori* eradication. **(A)** Frequency bursts of the 13 author keywords meeting the prespecified criteria. Burst detection was conducted using a Kleinberg-style multistate Poisson model over the complete publication years from 1993 through 2025. The light-blue line represents the complete observation period, and the orange segment represents the detected burst interval. Values on the right indicate burst strength and the corresponding start and end years. **(B)** The 15 cited references with the strongest citation bursts. Cited-reference identities were normalized across Web of Science and Scopus before analysis. The light-blue line represents the complete observation period, and the orange segment represents the burst interval. Values on the right indicate burst strength and the corresponding start and end years.

Supplementary Table S1 Complete search strategies for Web of Science, Scopus, and PubMed.

Supplementary Table S2 Deduplication, database coverage, screening workflow, exclusion reasons, and screening audit.

Supplementary Table S3 Software versions, data fields, counting methods, network thresholds, and final analytical parameters for main and supplementary figures.

Supplementary Table S4 Author-keyword co-occurrence clusters, representative terms, and interpretive research themes.

Supplementary Table S5 Author-keyword and cited-reference bursts in the primary complete-year analysis.

Supplementary Table S6 Clinical decision-domain codebook, reviewer agreement, temporal distributions, expected cell counts, statistical sensitivity analysis, and 95% confidence intervals.
